# Handling Partially Observed Trial Data After Treatment Withdrawal: Introducing Retrieved Dropout Reference‐Base Centred Multiple Imputation

**DOI:** 10.1002/pst.2416

**Published:** 2024-07-16

**Authors:** Suzie Cro, James H. Roger, James R. Carpenter

**Affiliations:** ^1^ Imperial Clinical Trials Unit Imperial College London London UK; ^2^ Medical Statistics Department London School of Hygiene & Tropical Medicine London UK; ^3^ MRC Clinical Trials Unit @ UCL UCL London UK

**Keywords:** Gaussian repeated measures, multiple imputation, off‐treatment, reference‐based, retrieved dropout

## Abstract

The ICH E9(R1) Addendum (International Council for Harmonization 2019) suggests treatment‐policy as one of several strategies for addressing intercurrent events such as treatment withdrawal when defining an estimand. This strategy requires the monitoring of patients and collection of primary outcome data following termination of randomised treatment. However, when patients withdraw from a study early before completion this creates true missing data complicating the analysis. One possible way forward uses multiple imputation to replace the missing data based on a model for outcome on‐ and off‐treatment prior to study withdrawal, often referred to as retrieved dropout multiple imputation. This article introduces a novel approach to parameterising this imputation model so that those parameters which may be difficult to estimate have mildly informative Bayesian priors applied during the imputation stage. A core reference‐based model is combined with a retrieved dropout compliance model, using both on‐ and off‐treatment data, to form an extended model for the purposes of imputation. This alleviates the problem of specifying a complex set of analysis rules to accommodate situations where parameters which influence the estimated value are not estimable, or are poorly estimated leading to unrealistically large standard errors in the resulting analysis. We refer to this new approach as retrieved dropout reference‐base centred multiple imputation.

## 1 Introduction

In pivotal clinical trials with longitudinal follow‐up, it is now common practice to attempt to collect full outcome data after the withdrawal of randomised treatment up until the nominal end of study. This allows the study to readily adopt a treatment‐policy strategy for handling treatment withdrawal, to target the effect of the randomised treatment regardless of any treatment non‐adherence [1]. As discussed by Wang et al. [2], for regulatory decision making a treatment policy strategy is most often utilised in order to estimate how well a treatment will work in clinical practice. This follows the intention‐to‐treat (ITT) principle. Henceforth, we denote the intercurrent event of randomised treatment withdrawal as *deviation* following Carpenter, Roger and Kenward [3]. If all post‐deviation data are collected estimation of an estimand using a treatment policy strategy will be straight forward; standard analytical methods (e.g., ANCOVA) can be applied to the observed data. However, after deviation, collection of complete data is often problematic. It is common for as many as half of those patients to have incomplete outcome data relevant to the estimand variable and summary measure. This may be by design, where less invasive monitoring is subsequently implemented, or due to a failure to implement observation despite best efforts, or because the patient withdraws further consent. Thus, most often, estimation requires appropriate assumptions for the unobserved post‐deviation data, which critically should align with the treatment policy strategy (i.e., reflect what actually happens to outcomes after treatment deviation). Fletcher et al. [4] on behalf of the EFSPI Estimand Implementation Working Group (EIWG) suggest that ‘there is little statistical literature concerning the unbiased estimation of estimands using the treatment policy strategy, and this is an area requiring significantly more focus and attention’.

There are typically two main pathways for deviating trial patients; either progress to a pre‐specified follow‐on treatment (or no‐treatment) and continue to be observed, or immediately leave the study with no further participation. There is also an important intermediate pathway where a patient leaves the study early while on the follow‐on treatment, generating partially observed off‐treatment data as discussed later. Usually, the follow‐on treatment will be the same for each arm, as otherwise this distinction becomes an essential part of the treatment comparison. This shared follow‐on treatment regime may be the same or similar to practice in the placebo or reference arm, suggesting that those who discontinue in the placebo arm will experience little or no change in outcome, although they will be aware that they are off treatment while still being unaware which treatment they had previously been having. But on the other hand, the follow‐on treatment may be less prescriptive than on‐treatment reference including scope for rescue. This aspect of the trial protocol is just as important when modelling off‐treatment outcomes as when interpreting the treatment‐policy estimand. When understood, it allows the generation of realistic models for unobserved outcomes during the follow‐on period.

Three main ways forward have been proposed for handling partially observed post‐deviation data in a longitudinal trial setting when adopting a treatment‐policy strategy, mostly based on multiple imputation. First to ignore compliance status and treat the data as a simple missing‐at‐random (MAR) missing data problem with the intercurrent event status ignored. However, this approach as we expand further on below is not fully aligned with a treatment‐policy strategy. Second, to use reference‐based imputation (RBI) and assume the deviators behave like those observed in a specified reference group of the trial (typically placebo/control) [3]. A similar approach that borrows data from elsewhere in the trial is return‐to‐baseline imputation whereby imputation is based on the patient's own baseline observation as mean. These approaches often ignore any off‐treatment data in the imputation stage and then merge any observed off‐treatment data back in. Wolbers et al. [5] discuss ways to integrate off‐treatment data from the reference arm into the imputation stage. Third, model the observed off‐treatment and on‐treatment data in a combined model and then impute the missing data based on this estimated model. Different variants of this latter approach, often referred to as retrieved drop out multiple imputation, use either imputation at final visit only, or a repeated measures based imputation across all visits, with the added complexity of differing choices for the repeated measures means model. These three ways forward and their variants are discussed in more detail in the following sections.

Approaches based around RBI disregard potential information about the outcomes off‐treatment during imputation and also make strong assumptions that may not always be consistent with trial experience in the active arm after treatment withdrawal. Retrieved drop out approaches based on modelling outcome after treatment withdrawal may be impractical because parameters are, or may be, poorly estimated due to limited observed off‐treatment data post‐deviation [2, 6]. Also, it may be difficult to pre‐specify an appropriate off‐treatment model that reflects the complexities while allowing the parameters to be reasonably well estimated.

In this article, we propose a novel multiple imputation approach that draws its influences from these two disparate approaches. The idea is to have a simple core model very similar to those used in RBI. There the parameters for the off‐treatment period are borrowed from those for the reference arm, usually placebo. This core model is then extended with a series of additional parameters so that the extended model is equivalent to the retrieved dropout type of model used for modelling off‐treatment outcomes as previously described. The extended model is fitted using a Bayesian framework with typically uninformative priors for the core model and mildly informative priors for the additional parameters. In this way when there is little observed off‐treatment data the subsequent imputed data will behave somewhat similar to RBI but with some slight increase in variation representing uncertainty about the RBI assumption. Indeed, some will see this as an improvement over RBI with its total acceptance for the RBI's assumptions. On the other hand, when there is a large proportion of observed off‐treatment data available this will over power the mild priors and the imputation will behave like the chosen off‐treatment model. We refer to this new approach as retrieved dropout reference‐base centred multiple imputation.

This general concept can be applied to different kinds of outcome data including binary, counts, time‐to‐event as well as general quantitative outcomes. For simplicity of description, in this paper we focus on the case of a classic repeated measures multivariate normal model, with two treatment arms and possible baseline measurements to be treated as covariates. The concept of using mildly informative priors for a subset of parameters as a safety net should they be poorly supported by the actual data, could similarly be used to handle different intercurrent events using a treatment policy strategy, such as temporary treatment interruptions or rescue medication. However, choice of parameterisation and possible dependence between parameters in the prior would need careful specification influenced by clinical expertise and we focus on treatment withdrawal (denoted deviation) throughout.

In Section 2, we describe two motivating trial data sets, which have identical data where observed as well as the same level of off‐treatment data post‐deviation, but with missingness distributed differently between arms. The goal is to address an estimand using a treatment policy strategy to handle treatment deviation, but one data set is problematic while the other is not. We then review the current methods to handling partially observed treatment withdrawal data in the context of estimation for a treatment policy strategy in more detail in Section 3. Our novel retrieved dropout reference‐base centred multiple imputation approach, which builds upon the current methods, is described in full in Section 4. In Section 5, we apply our new method and existing methods to the two example data sets and finish with a discussion in Section 6.

## 2 Example Data Sets

To illustrate our approach, we explore two publicly available example data sets based on real underlying data from an anti‐depression trial, originally conducted by Goldstein et al. [7], with the addition of simulated partial off‐treatment data. The trial's primary outcome is change from baseline in Hamilton 17‐item rating scale for depression (HAMD17) which was measured at baseline, 1, 2, 4, 6 and 8 weeks. Mallinckrodt et al. extracted the on‐treatment data from the original trial including a placebo group and an active group randomly chosen from three non‐placebo arms: two doses of an experimental medication and also a medication approved at the time [8]. Data for the final visit, Week 8, which was the original primary endpoint were not included and have never been made publicly available. For the purpose of this analysis the Week 6 endpoint and completion status define the estimand of interest. Completion rates (on‐treatment) were 76% (64/84) for active and 74% (65/88) for placebo and the profile of visit‐wise mean changes for patients who completed the trial versus those who discontinued early are summarised in fig. 1 of Mallinckrodt et al. [8]. Michael O'Kelly and Sylvia Li extended this data set with simulated off‐treatment data while exploring the properties of differing analysis methods and made available template SAS code through the Drug information Association Scientific Working Group on Estimands and Missing Data (SWGEMD) [9]. Deposited with the templates and freely available for download are the two data sets chosen to illustrate the methods at work and also to indicate the problems that occur when parameters in the model are non‐estimable [9].

For both data sets, the estimand of interest is the mean difference in the change in HAMD17 at Week 6 (Visit 4) among the eligible trial population with depression for active treatment versus placebo regardless of whether all treatment was received (treatment policy). Four patients had baseline less than 7, while 77 were in the mild range (7–17), 73 in the moderate range (18–24) and 18 severe (over 24). HAMD17 scores are expected to fall over the 6 weeks, indicating improvement, even in the placebo arm. The final column for placebo in Tables 2 and 3 shows means for those who complete the trial on‐treatment, with a reduction in mean of about one unit per visit. A larger drop of about 2 units per visit is seen for the completers in the active arm. On‐treatment data are fully observed and both data sets have the same number of on‐treatment patients at each visit in each arm. Off treatment data were simulated using a model estimated from the control group data only, conditional on a patient's baseline attributes (baseline score and a pooled‐site indicator) and also their observed on‐treatment post‐baseline outcomes. The extent of missing off‐treatment data is summarised in Table 1 with all patients on treatment at the first visit. In these data sets all patients who deviate either stop follow‐up immediately or continue to the end of the study period with complete observed off‐treatment data. The first labelled *converging* by the original authors is called *covered* here to indicate that it contains data which ‘cover’ all subgroups defined by the data generation models. As shall be later demonstrated this data set works well with all the multiple imputation approaches explored in this article. The second labelled *nonconverging* by the original authors and called *perforated* here has no observations after deviation for any of the six patients in the active arm who deviate at Week 2. This results in non‐estimable parameters for some of the models discussed later. The *perforated* data set has the missingness arranged in such a way as to generate a hole in the data. Since the on‐treatment data in both data sets are based on the same original underlying data set, similar but not identical results are to be expected. Tables 2 and 3 summarise the means by visit for each combination of treatment, deviation visit and presence of off‐treatment observation in the two data sets. In essence to estimate the treatment‐policy estimand the blank cells in the Visit 4 row need to be filled out and then the difference in means taken across columns for placebo, and then active, weighted by the count row.

**TABLE 1:** Summary of on‐treatment, observed off‐treatment and unobserved off‐treatment frequencies by visit in example data sets.

	*Covered*						*Perforated*					
	Placebo			Active			Placebo			Active		
Visit	On	Off‐Obs	Off‐Miss	On	Off‐Obs	Off‐Miss	On	Off‐Obs	Off‐Miss	On	Off‐Obs	Off‐Miss
1	88	0	0	84	0	0	88	0	0	84	0	0
2	81	3	4	78	4	2	81	5	2	78	0	6
3	76	7	5	73	5	6	76	9	3	73	4	7
4	65	12	11	64	10	10	65	12	11	64	10	10

**TABLE 2:** Summary of on‐treatment (non‐bold) and observed off‐treatment means (bold) by pattern of deviation and missingness in *Covered* data set.

	*Placebo*							*Active*						
	Last visit on treatment							Last visit on treatment						
	1		2		3		4	1		2		3		4
Visit	Stop	Cont.	Stop	Cont.	Stop	Cont.	Cont.	Stop	Cont.	Stop	Cont.	Stop	Cont.	Cont.
1	1.25	−1.67	−6.00	−0.50	−1.00	−0.20	−1.82	−2.00	1.25	−2.00	−10.00	−1.75	−3.40	−1.75
2		**−2.29**	−4.00	2.00	−2.83	−0.80	−3.11		**0.19**	−4.00	−4.00	0.00	−5.20	−4.87
3		**−4.80**		**−0.97**	−2.67	−0.80	−4.45		**−2.71**		**−5.00**	−0.50	−6.00	−7.25
4		**−4.21**		**−1.32**		**−2.44**	−5.14		**−2.30**		**−7.01**		**−6.39**	−8.34
Count	4	3	1	4	6	5	65	2	4	4	1	4	5	64

*Note*: Patients either stop follow‐up at the deviation or continue being observed while off treatment until the end of trial.

**TABLE 3:** Summary of on‐treatment (non‐bold) and observed off‐treatment means (bold) by pattern of deviation and missingness in *Perforated* data set.

	*Placebo*							*Active*						
	Last visit on treatment							Last visit on treatment						
	1		2		3		4	1		2		3		4
Visit	Stop	Cont.	Stop	Cont.	Stop	Cont.	Cont.	Stop	Cont.	Stop	Cont.	Stop	Cont.	Cont.
1	−1.00	0.40	−6.00	−0.50	0.13	−2.67	−1.82	0.17		−5.00	−3.25	−0.67	−3.67	−1.75
2		**−0.67**	−4.00	2.00	−0.75	−5.00	−3.11			−7.00	−3.25	−1.33	−3.67	−4.87
3		**−2.93**		**−0.97**	0.00	−6.67	−4.45				**−4.18**	0.33	−5.50	−7.25
4		**−2.97**		**−1.32**		**−7.25**	−5.14				**−5.29**		**−6.20**	−8.34
Count	2	5	1	4	8	3	65	6	0	1	4	3	6	64

*Note*: Patients either stop follow‐up at the deviation or continue being observed while off treatment until the end of trial.

## 3 Current Methods

In this section, we now review in more detail the three main approaches proposed to date for estimating an estimand with a treatment policy strategy for handling deviation with missing post‐deviation data.

### 3.1 Ignoring Compliance

The first approach assumes all outcomes are MAR (i.e., can be predicted based upon modelled covariates and observed outcomes) ignoring whether a patient is on‐ or off‐treatment. As the majority, if not all, of the missing data is off‐treatment, imputation using a combination of on‐treatment and off‐treatment data by ignoring compliance is likely to be biased. Guizzaro et al. [10] used causal inference methods to indicate the importance of including an indicator for compliance in the imputation model when a treatment policy strategy is implemented and the intercurrent event of discontinuing treatment is not MAR.

Despite its inherent failings this MAR approach has been implemented using a classic MMRM analysis either by maximum likelihood or multiple imputation using Rubin's rules for inference [9, 11, 12, 13]. Since there is an incoherence in modelling off‐treatment data based on a mixture of on‐ and off‐treatment data for a treatment policy strategy it is generally unacceptable [14]. It should only be used in situations where very few missing data are expected to avoid deviating from meaningful estimation. However, there is no consensus on what constitutes very few. This use of on‐treatment data to estimate off‐treatment experience will be especially problematic when off‐treatment patient care allows wider treatment options such as novel treatments. The remaining approaches incorporate the compliance aspect in a series of different ways.

### 3.2 Reference‐Based Multiple Imputation

The second broad approach imputes all post‐deviation data using a reference‐based multiple imputation (RBI) method such as jump‐to‐reference (J2R), ignoring some [5] or all of the off‐treatment data and then, where available, replaces imputed values with actual observed measurements [3]. The underlying model for reference‐based multiple imputation of a continuous outcome is the standard repeated measures multivariate normal model including the treatment by time interaction, along with other covariates, which may or may not be crossed with time, and an unstructured covariance matrix that may be shared or separate for each arm. For imputation, a model is constructed for each pattern of deviation using the parameters based upon the underlying fitted multivariate normal model. How the construction is performed defines the different reference based methods. Multiple imputations are then drawn following the Bayesian paradigm. As in standard MAR MI, post imputation each imputed data set is analysed with the substantive analysis model of interest and results combined using Rubin's rules.

J2R imputation forms the imputation model using the same mean profile up to deviation as observed for the randomised arm, but post deviation the profile jumps to the estimated profile for the reference arm. Other reference‐based approaches include copy increments in reference (CIR) which forms the imputation model using the same mean profile up to deviation as observed for the randomised arm, but post deviation the profile tracks parallel to the pattern for a specified reference arm. It is also possible to implement different reference based assumptions for different individuals in the same trial based on reason for deviation. Polverejan and Dragalin [11] used CIR reflecting their interest in a long term degenerative disease where the effect of treatment will remain and there is no bounce back to untreated values as in J2R. This they label CIR in contrast to an analysis where the reference‐based assumption varied by type of deviation (labelled BY_REASON) where copy reference (CR, which uses the mean profile from the reference arm throughout) is used for AEs, J2R for loss of efficacy and CIR is used for the ‘other’ category. Both the EIWG [12] and Wang and Hu [15] use J2R as the RBI method when evaluating methods to target a treatment policy estimand, and there are now examples of this method being used in practice [16].

A variant of this method, referred to as the ‘return to baseline approach’ [2], assumes there is no placebo effect and any intervention effect will be washed out after deviation. Data are multiply imputed based on the mean baseline values [17]. This approach washes out any drug effect after treatment deviation while assuming any post‐deviation treatment prevents further deterioration from baseline. This can be viewed as a particular type of reference based multiple imputation where the reference is now the baseline mean. This contrasts with the baseline observation carried forward (BOCF) approach where the baseline observation is simply carried forward; this latter approach is not recommended since it underestimates within patient variability of the measurements at different time points [18]. Others have suggested imputing based on the patients baseline observation plus some random variation [2], but this has shown to result in biased mean when the missingness depends on observed baseline and/or post‐baseline intermediate outcomes and the variance of imputed values can be much larger than the variance of the baseline values [18].

A more simplistic reference‐based approach termed the ‘washout method’ by Wang et al. [2] excludes intermediate measurements in the imputation model. An imputation model is built based on placebo/reference completers only and the missing observations for those in the active arm at the primary endpoint are directly imputed without imputing any intermediate values. For patients in the placebo/reference arm the MAR assumption is accepted and intermediate outcomes are included in the imputation model. As described by Wang et al. [2] this approach washes out the experimental treatment effect (TE) in those who dropout after treatment deviation in the experimental/active treatment group. As Wang et al. [2] suggest as an aside in their appendix ‘the washout approach can be thought as a simplified version of the J2R approach without considering the intermediate measurements from any arms in the imputation’.

### 3.3 Retrieved Dropout Multiple Imputation

Here prediction after deviation relies directly on the observed data from those who deviate. A basis for such an approach is that prediction after deviation should rely only on observed data from those who deviate. The imputation model is either univariate predicting outcome at last visit, or uses a repeated measures model based on all visits involving compliance status in some form. Guizzaro et al. [10] indicate that their simulation ‘results suggest that at least in a “Missing at Random” setting, all studied estimation methods increase their performance when a variable representing compliance is used’. This important result was the purpose of their study rather than to compare their wide range of different methods that included three disparate forms of multiple imputation (predictive mean matching [PMM], classification and regression tree [CART] and Schafer's linear regression), inverse probability weighting and maximum likelihood using auxiliary variables. The approaches discussed in this section fall within the Schafer's linear regression method.

#### 3.3.1 Retrieved Dropout Compliance Model—Last Visit Only

Wang and Hu [15] propose a novel approach using multiple imputation of the outcome variable at final visit imputed for those who terminate prior to final visit without imputing earlier missing data. This imputation uses a regression for final outcome on patient's baseline and their last on‐treatment value, stratified by randomised treatment and the visit number of last on‐treatment visit. Intermediate outcome measurements either before or after the last on‐treatment visit are ignored, as well as ignoring those who complete on treatment.

An alternative approach, (the fourth approach in the SWGEMD templates [9], labelled A4) also imputes missing values at the final visit ignoring those who complete the trial on treatment. It assumes that the change in outcome from time of discontinuation of treatment to end of scheduled follow‐up is MAR in patients who discontinue study treatment, conditioning only on baseline, last on‐treatment outcome, and the length of time on study treatment. The time on study treatment is considered continuous and the relationship between time on study treatment and outcome is considered linear. This is then similar to Wang and Hu [15] but rather than stratify by treatment and off‐treatment visit number they regress on the numeric value of the visit (stratified by treatment only). This reduces the number of required parameters in the regression.

Focusing on imputation at the final visit alleviates the need for imputing intermediate missing values, but requires regressing on data that is certainly observed, such as the outcome at or just before deviation irrespective of when during the trial deviation occurred. This approach obviously suits those studies where after treatment withdrawal outcome is only measured at the final nominal visit. Also, the details of the imputation process are likely to be less contentious. But the strong assumption of linearity between outcome and time on study may not always be appropriate. Further, when intermediate off‐treatment data are extensive, such as in long term diabetes studies, there will presumably be some loss of information in discarding the intermediate data.

#### 3.3.2 Retrieved Dropout Compliance Model—Repeated Measures

Here the outcome is modelled across all visits accounting for compliance using all the observed data and some form of repeated measures model.

The simplest approach is to fit a MMRM model under MAR (see Section 3.1) and add a single indicator variable that denotes whether the patient discontinues treatment before the end of trial or not. Polverejan and Dragalin [11] denote this MAR_DC and also do the same ignoring the patients who complete denoted RD_SUBSET. The indicator variable On–Off(Patient) is defined at the patient level and denotes discontinuation of treatment at any time before the final visit.

At the other extreme, the most complex repeated measures model effectively stratifies by both treatment arm and the index of the last visit on treatment and imputes within each stratum combination [2]. The second approach in the SWGEMD templates (labelled A2) does something very similar but while stratifying by treatment it does not cross baseline with treatment. Instead, it uses a sequential regression with baseline and an interaction between treatment and index of last visit on treatment. In the former purest form of complete stratification by arm, the study completers who are still on‐treatment are irrelevant in the imputation process as they form their own stratum with no missing observations; here their data does not inform the imputation of the unobserved off‐treatment data. This pure approach is MI3 from EIWG [12] and M4 in Roger [13] (see Table 4). As implemented by SWGEMD (A2) the completers have impact on estimated baseline regression coefficients. All the implementations effectively use a single covariance matrix with no attempt to fit separate covariance matrices either by arm or, by before‐deviation versus after‐deviation as in Carpenter, Roger and Kenward [3]. Both these models have the property that means differ depending upon whether the patient deviates or not at later visits.

**TABLE 4:** Compliance based models, using retrieved dropout data, for handling partially observed data after treatment withdrawal.

Current^a^	Historic^b^	Full‐Pattern^c^
$E\left[Y_{\mathrm{tj}}\right]$ = $\mu_{\mathrm{tj}}$ for j≤k	$E\left[Y_{\mathrm{tj}}\right]=\mu_{\mathrm{tj}}$ for j≤k	$E\left[Y_{\mathrm{tj}}\right]=\gamma_{\mathrm{tkj}}$ for all j,k
$E\left[Y_{\mathrm{tj}}\right]=\alpha_{\mathrm{tj}}$ for j>k	$E\left[Y_{\mathrm{tj}}\right]=\gamma_{\mathrm{tkj}}$ for j>k	
$\alpha_{\mathrm{tj}}$ is the mean at visit *j* for the stratum defined by treatment *t* and being off treatment at visit *k*	$\gamma_{\mathrm{tkj}}$ is the mean at visit *j* for the stratum defined by treatment *t* and the last‐on‐treatment visit *k* (history)	$\gamma_{\mathrm{tkj}}$ is the mean at visit *j* for the stratum defined by treatment *t* and the last‐on‐treatment visit *k* (history)

*Note*: For a continuous outcome $Y_{\mathrm{ij}}$ measured for patient i at times j=1,…,J, where k is the last observation time prior to treatment discontinuation (i.e., deviation) and $\mu_{t,j}$ is the mean for treatment arm t at time j.

^a^ Referred to as A3 by SWGEMD [9], M12 by EIWG [12], M2 by Roger [13] and rd_trt and rd_trt_dcreason by Polverejan and Dragalin [11].

^b^ Referred to as A2a by SWGEMD [9] and M3 by Roger [13].

^c^ Referred to as A2 by SWGEMD [9], M13 by EIWG [12] and M4 by Roger [13].

If there are J visits in the later A2 model, then potentially $J^{2}$ parameters are introduced into the model, *J* for each regression, although in the purest form many of these have no influence in the imputation process. For large *J* this can be problematic. An adapted version of their second approach, A2a, is proposed within the SWGEMD code [9] where each regression only depends on ‘last visit’ number up to the current one with all those reaching this far on treatment pooled in one level (see Table 4). This is equivalent to the M3 method in Roger [13]. Effectively the strata or factor levels are defined by pooling all those patients who are on treatment at this visit. Indeed, if baseline were absent from the model crossed with treatment, then this pooling would have no impact on the subsequent imputation. Whether pooled or not the series of factors are nested within each other from regression to regression allowing sequential regressions to be based on either previous observed or residual outcomes. This A2a approach we will call the *historic* compliance model as it accounts fully for the compliance history so far as a whole, in contrast to the A2 approach described previously which we refer to as *full‐pattern*.

All the same, the final regression will potentially have *J* strata and some of these may be very small with little outcome data observed at final visit. Whenever the strata size are small (i.e., few individuals with the specific deviation pattern) or study completion rate is low, sharing information between strata should improve precision at the potential expense of introducing bias.

A simpler approach, uses an on/off treatment indicator at each visit within each arm but unlike the first approach this indicator changes value from visit to visit and hence from regression to regression [19]. This means that the model at each visit is not nested within the model at subsequent visits as the variable for on/off treatment changes from visit to visit. This is the third approach labelled A3 in the SWGEMD templates [9] (see Table 4). It is similar to proposed methods RD_TRT and RD_TRT_DCREASON discussed in Polverejan and Dragalin [11], MI2 form EIWG [12] and M2 in Roger [13]. In order to fit this model as a sequence of regressions each regression in the sequence needs to regress on previous residuals rather than on previous observed outcome values. For example, in the SWGEMD SAS code this is done using an intermediate call to the MIXED procedure between calls to the MI procedure, or using the MISTEP macro from the DIA missing data website [9]. The simplifying assumption in this model is that the marginal impact of being off treatment at the current visit is the same whether or not you were off treatment at previous visits. We refer to this A3 approach as the *current* compliance model as it accounts for whether or not the patient is currently on treatment.

A relatively large reduction in number of parameters can also be achieved by regressing on the quantitative length of time on treatment rather than handling deviation visit as a multilevel factor. This is implemented in the SMWEG A4 template code, which is discussed in the above section [9].

In general, it has been shown that analyses ignoring compliance (i.e., on/off treatment status in any form) can introduce substantial bias and can sometimes underestimate variability [6, 19, 20]. While retrieved dropout compliance MI models can successfully reduce or correct the bias, they inevitably lead to increases in variance which can be substantial if most (i.e., ≥50%) post‐deviation data is missing [6].

## 4 Retrieved Dropout Reference‐Base Centred Multiple Imputation

We now describe our novel approach which combines a core reference‐based model with a compliance model using retrieved dropout data to form an extended model for the purposes of imputation. Assume a total of *N* patients are randomised to two treatment groups, reference and active. They are observed at baseline and *J* subsequent visits, with interest in the outcome at the final visit *J*. Let $Y_{i}=\left(Y_{i1},\ldots ,Y_{\mathrm{iJ}}\right)$ denote the longitudinal vector of continuous outcome values for the *i*th patient and $T_{i}$ denote randomised arm taking values 0 for reference and 1 for active. For simplicity, assume there are no interim missing data and all patients are observed at baseline and up to visit $D_{i}$, the last observation time prior to treatment discontinuation for patient i, known as deviation here. This is set to final visit J for those completing on‐treatment. Patients who withdraw from treatment and also leave before the end of the study having no final visit outcome measure, have their final visit observed within the study at visit $S_{i}$, which is set to J for those completing study. Patients halt study treatment when leaving the study and so $S_{i}\geq D_{i}$ for all patients. Outcome values off‐treatment are assumed to be available between visits $\left(D_{i}+1\right)$ and $S_{i}$ inclusive for the *i*th patient but missing after $S_{i}$. This set will be null when deviation and study withdrawal concur ($D_{i}=S_{i}$). The on‐treatment model conditional on remaining on treatment has mean outcome $E\left[Y_{\mathrm{ij}}|D_{i}\geq j,T_{i}=t\right]=\mu_{\mathrm{tj}}$.

Two RBI methods are commonly used and offer possible core models [3]. First J2R is defined as

(1) $$\begin{array}{rr} E\left[Y_{\mathrm{ij}},|,D_{i}=k,,,T_{i}=t\right] & =\mu_{\mathrm{tj}}\;\text{for}\;j\leq k\\ & =\mu_{0j}\;\text{for}\;j>k \end{array}$$

while CIR is defined as

(2) $$\begin{array}{r} E\left[Y_{\mathrm{ij}}|D_{i}=k,T_{i}=t\right]=\mu_{\mathrm{tj}}\;\text{for}\;j\leq k\\ \;=\left(\mu_{0j}+\mu_{\mathrm{tk}}-\mu_{0k}\right)\;\text{for}\;j>k \end{array}$$

J2R is suited to a treatment that is expected to have only short term effect and where the response would return to that of the reference after treatment withdrawal. On the other hand, CIR is more appropriate for a treatment where the improvement obtained from treatment up to time of deviation will continue, but further improvement relative to reference is unlikely. An intermediate profile where an explicit assumption is made about how the maintained effect of treatment after discontinuation relates to the effect of treatment before discontinuation using the approach of White, Joseph and Best [21] may alternatively be used for the core mean model, or another shape based on the reference arm experience. Three other potential core models are last mean carried forward (LMCF), where the mean stays the same as the last on‐treatment mean for a patient, and return to baseline (RTB) where the off‐treatment mean is the baseline mean within arm or across both arms, or MAR where the means are simply $E\left[Y_{\mathrm{ij}}|T_{i}=t\right]=\mu_{\mathrm{tj}}$. The core model could also include a fixed ‘delta’ depending on visit or number of visits since deviation as demonstrated in one of the later examples below. It is important that the choice of core model is based on clinical experience and plausibility, rather than mathematical simplicity.

Note how the on‐treatment parameters $\mu_{\mathrm{tj}}$ can be estimated from on‐treatment data and the means for the off‐treatment period via the core model (RBI or other) conditional upon the history (on/off treatment pattern so far). When data are available for the off‐treatment period it is possible to fit a more complex model for this period (for j>k) by also using one of the suggested compliance models. This we call the *extended* model. The idea is to centre the extended model on the core model by simply subtracting off the projected means from the core model. Then if the core model is a good description, the extra deviation part (difference between compliance model and core model) will be small in value. When we expect holes in the data one way forward to make the compliance model, hence deviation parameter, estimable is to use a mildly informative zero‐centred prior for the extension parameters. However, the core model, which may condition on the history (on/off treatment pattern so far) such as with CIR, needs to be nested within the compliance model otherwise it will distort the resulting compliance model. That is when sufficient off‐treatment data are available and the prior is made totally uninformative the compliance model ought to be recovered as a special case. Examples where nesting does not hold are discussed in the following section.

There is some choice for the compliance part of the extended model with the saturated compliance model adding a treatment by history by visit interaction in the off‐treatment period.

(3) $$\begin{array}{r} E\left[Y_{\mathrm{ij}},|,D_{i}=k,,,T_{i}=t\right]=\mu_{\mathrm{tj}}\;\text{for}\;j\leq k\\ \;\;=\gamma_{\mathrm{tkj}}\;\text{for}\;j>k \end{array}$$

where $\gamma_{\mathrm{tkj}}$ is the mean at visit j for the stratum defined by treatment *t* and the last‐on‐treatment visit k (history). This is the *historic* compliance model, Pattern * Treatment * Visit, and is preferred in this context to the *full‐pattern* compliance model, Subject_Pattern * Treatment * Visit, where Subject_Pattern is declared at the subject level and reflects the compliance throughout the trial. In contrast the Pattern only reflects the compliance so far. There are P+1 levels for both Pattern and Subject_Pattern, the visit number of the last visit on treatment, the difference is that Pattern is set to the current Visit number while on treatment, so that Pattern * Visit has potentially $P^{*}\left(P+1\right)/2$ levels while Subject_Pattern * Visit has potentially the full $P^{*}\left(P+1\right)$ levels.

Formally, the proposed approach is to re‐parameterise this extended model by including an additional offset term determined by a reference‐based model for the post deviation means. No additional parameters are needed as the reference based mean involves parameters μ from the on‐treatment observations, and these parameters concur with those for the extended model. What changes is the interpretation of the parameters in the extended model. These are now deviations from the reference‐based mean rather than from a global mean of zero. For instance, when J2R is used as reference‐based core the extended model becomes
(4)

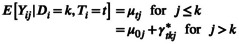

Then a mildly informative zero‐centred prior is applied to the parameters $\gamma^{*}$ while retaining an uninformative prior for μ (the parameters from the core model). Note how in Equation (4), the same parameters μ are shared between the on and off‐treatment periods by the J2R RBI model. There is no aliasing of parameters between μ and $\gamma^{*}$ as $\mu_{0j}$ for j>k is inherently estimated by the data where j<k in other patients. When data are not available for the off‐treatment period then in expectation this reduces to the J2R reference based imputation as the $\gamma^{*}$ priors have zero mean. This complex extended model would suit any other core model such as CIR equally well. When off‐treatment data are available then $\gamma^{*}$ becomes estimable and in the Bayesian model some very small amount of information will be fed back into μ as $\gamma^{*}$ has a mildly informative prior. However, this is not an issue as the model is only being used to impute missing data in the off‐treatment region. Any intermediate missing values prior to deviation are treated as MAR.

Less flexible options for the extended model could be chosen based on experience with modelling retrieved dropout. An alternative simple choice is to replace the parameters $\gamma_{\mathrm{tkj}}$ by $\alpha_{\mathrm{tj}}$ and $\gamma_{\mathrm{tkj}}^{*}$ by $\alpha_{\mathrm{tj}}^{*}$ in the same way as the *current* compliance model based on On–Off(Visit) * Treatment * Visit where On–Off(Visit) is a visit level indicator for whether the patient is on or off treatment.
(5)

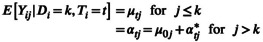

Here $\alpha_{\mathrm{tj}}^{*}$ is the mean at visit *j* for the stratum defined by treatment *t* and the impact of being off‐treatment on the expected outcome is the same however long the patient has been off‐treatment. Note how Off(Visit) * Visit has only 2P potential levels. Replacing the interaction in the full‐history model by two main effects is another option. Linearisation of γ or $\gamma^{*}$, rather than using a distinct mean for each visit (in a similar way to A4 in SWGEMD [9]), is a further possible simplification but neither is explored here.

### 4.1 Illustration in a Simple Setting

We now illustrate our proposal, and show how it relates to the methods described in Section 3, using a simple two‐arm trial setting with no baseline measurement and a single outcome indicated generally by *Y*. As above, subscript 0 is used to denote reference arm and subscript 1 is used to denote active arm.

Suppose a proportion $p_{1}$ have experienced deviation and ceased treatment before outcome measurement in the active arm. These experience off‐treatment behaviour. Of those off‐treatment, $q_{1}$ are not observed for their outcome. So of all patients in the active arm, the proportion $p_{1}q_{1}$ are not observed off‐treatment while $p_{1}\left(1-q_{1}\right)$ are off‐treatment but observed, and $\left(1-p_{1}\right)$ are observed on‐treatment. All patients on‐treatment are observed. The outcome is $y_{\text{miss},1}$ for those not observed, $y_{\mathrm{off},1}$ for those observed off‐treatment and $y_{\mathrm{on},1}$ for those observed on‐treatment in the active arm. This forms three groups indexed by j=miss,off,on. The $y_{\text{miss},1}$ values are never known. In the reference arm we assume for this illustration all individuals are observed on reference treatment. In Appendix 2 (and in Section 4.3.1), additional treatment deviation and missingness in the reference arm is explored.

We assume the outcome is distributed Normally with mean $\mu_{1}$ with variance $\sigma^{2}$ in the active arm and distributed Normally with mean $\mu_{0}$ with variance $\sigma^{2}$ in the reference arm. In the active arm patients are indexed within group by $i=1,\ldots ,n_{j,1}$ where $n_{j,1}$ is the total number observed in group *j*. Note that $n_{j,1}$ is not fixed but varies at random across repeat trials.

The TE contrast of interest is,

$${\mathrm{TE}}_{\text{Real}}=\left[\left(1-p_{1}\right)\mu_{\mathrm{on,}\;1}+p_{1}\left(1-q_{1}\right)\mu_{\mathrm{off,}\;1}+p_{1}q_{1}\mu_{\text{miss},\;1}\right]-\left[\mu_{0}\right]$$

the difference in expected value in each arm.

Patients are assumed independent, so that in the active arm conditional on which of the three groups a subject falls into, their outcome is sampled from a normal distribution with mean that depends on the treatment arm and whether the subject is observed and on‐ or off‐treatment. That is conditional on the number observed/deviating there are four means $\mu_{\text{miss},1},\mu_{\mathrm{off},1},\mu_{\mathrm{on},1},\mu_{0}$ and without further constraint $\mu_{\text{miss},1}$ is not in practice estimable. An obvious simple compliance model in this setting sets $\mu_{\text{miss},1}=\mu_{\mathrm{off},1}$ for active where the off‐treatment mean is the same whether observed or not, assuming generally MAR for the missing process (note in this simple example MAR and MCAR are equivalent as no additional variables, e.g., baseline covariates or observed previous outcome are being conditioned upon). Then we can estimate all four means as long as the patient counts for each mean are greater than zero. And we can estimate the contrast ${\mathrm{TE}}_{\text{Real}}$ as long as count $n_{\mathrm{off},1}>0$ whenever $n_{\text{miss},1}>0$. Under this MAR assumption,

$${\mathrm{TE}}_{\text{Real}}=\left[\left(1-p_{1}\right)\mu_{\mathrm{on,}\;1}+p_{1}\mu_{\mathrm{off,}\;1}\right]-\left[\mu_{0}\right]$$

A RBI J2R model uses $\mu_{\text{miss},1}=\mu_{\mathrm{off},1}=\mu_{0}$ instead, using the reference arm on‐treatment mean for the off‐treatment mean in the active arm, and does not in general require $n_{\mathrm{off},1}>0$ unless $n_{\text{miss},1}>0$.

The novel reference‐base centred proposal re‐expresses a compliance model for the active arm in the following way, $\mu_{\text{miss},1}=\mu_{\mathrm{off},1}=\mu_{0}+\gamma_{1}$. This is equivalent to the compliance model using MAR for the missingness process. When $\gamma_{1}$ is fixed at zero then this is the J2R RBI model. If $n_{\mathrm{off},1}=0$ while $n_{\text{miss},1}>0$ then it is the parameter $\gamma_{1}$ which cannot be estimated. But, by applying multiple imputation and using a mild informative zero‐centred prior for $\gamma_{1}$ the four means can all be estimated, leading to an estimate for the required treatment contrast as long as $n_{\mathrm{on},1}>0$.

### 4.2 Generic Method

To summarise, the generic algorithm of the proposed retrieved dropout reference‐base centred multiple imputation approach is as follows,

1. Choose (i) a required retrieved dropout compliance model (for example, historic or control) and (ii) a core reference‐based model (e.g., J2R/CIR/LMCF) and check this is nested in the selected compliance model.
2. Parameterise the compliance model for the means using two sets of parameters, the first representing the core RBI model and the second involving additional parameters that complete the compliance model. When the additional parameters are all set to zero the subsequent model is identical to the RBI core model.
3. Fit this as a Bayesian repeated measures multivariate normal model, taking all observed data, including treatment and the indicators required for the chosen compliance model with an unstructured covariance matrix (which could be grouped by treatment arm if required by the compliance model). Any baseline covariates could also be added as required. Use uninformative priors for the core mean and covariance matrix parameters. Use informative priors for the additional mean parameters (see Section 4.3).
4. Take a random draw from the posterior for all parameters.
5. As in standard MI, for each patient who deviates and has missing data before the end of the study, use the parameter values drawn in Step 4 to predict their missed values based on that patients' conditional distribution of post‐deviation data given their pre‐deviation data. Use these imputed outcomes to form a complete data set.
6. Repeat Steps 4 and 5 K times, resulting in K imputed data sets.
7. Fit the model of interest to each imputed data set. This will usually be a univariate model to estimate the estimand of interest, such as ANCOVA.
8. Merge the results across imputed data sets using Rubin's rules (1987) for final inference.

Appendix 1 shows how to implement this general approach in SAS using the BGLIMM procedure. The MCMC algorithm implemented by BGLIMM does the above Steps 4 and 5 readily in one step, as the imputations are synonymous with the estimated missing data parameters. This relies on the fact that the parameter estimation and the imputation models are identical.

### 4.3 Choice of Prior

The variance chosen for the prior for the additional deviation parameters will alter results, varying the underlying assumptions between the RBI core and the full compliance model as extremes, and therefore needs to be carefully considered.

We consider three potential routes for choosing the prior's variance for the additional deviation parameters. First, get experts together and illicit how far they expect the underlying compliance model might diverge from the chosen core model. Second, express the prior in terms of the effective number of patients being added into the data to modify the likelihood in this way, for example one or two extra patients worth of data who follow the core model exactly may be reasonable. Note that these extra subjects do not impact on the core model parameters, only on the additional parameters reflecting how far the compliance model may deviate from the core model. Following this second route, one proposal is to use the residual variance of the outcome at final visit from a simple MMRM model of on‐treatment data as a basis. Assuming that each of the additional parameters represents a deviation in predicted outcome between the core model and the more flexible compliance model, then a zero mean prior with this variance is roughly equivalent to providing one extra patient‐visit worth of additional likelihood into the posterior. Alternatively, one might use the normal or full range of the outcome as a simple starting point, for example, the square of one sixth of the range as variance. Third, use simulation to quantify the impact of the priors under differing amounts of deviation and missingness in the two arms designed around a compliance model based on previous experience. Then choose suitable priors that match the objectives of the trial in terms of; controlling uncertainty from potentially missed data, overt control of variation (SE for contrast) through informative priors, and unreasonably strong use of assumptions inherent in the core model. In practice we expect researchers would use a combination of these approaches.

#### 4.3.1 Investigating the Impact of the Choice of Variance for the Prior

**FIGURE 1:**
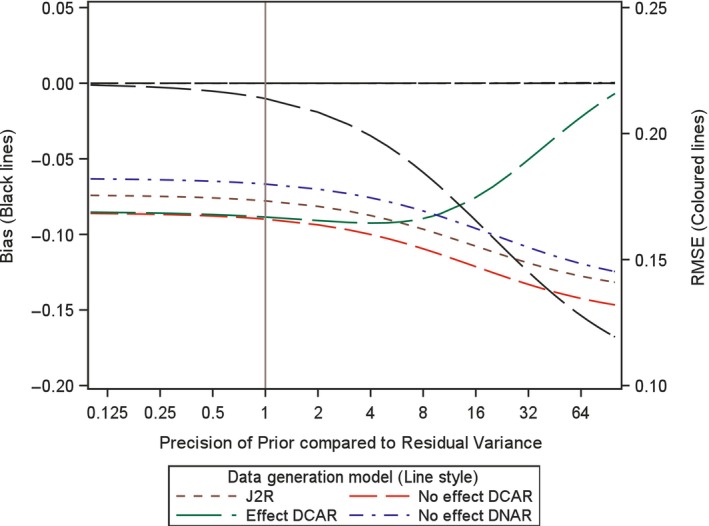
Bias and root mean squared error (RMSE) for retrieved dropout reference‐base centred multiple imputation with *n* = 100 per arm with 40% deviation and 50% missing data in both arms for different dispersion of prior and differing data generation models.

**FIGURE 2:**
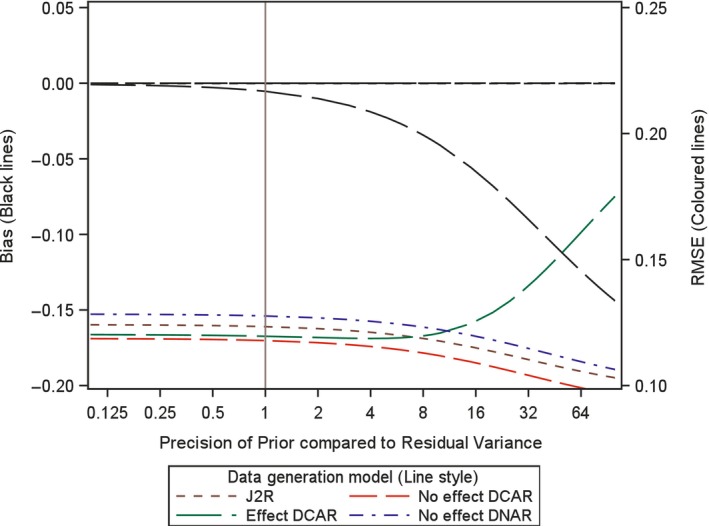
Bias and root mean squared error (RMSE) for retrieved dropout reference‐base centred multiple imputation with *n* = 200 per arm with 40% deviation and 50% missing data in both arms for different dispersion of prior and differing data generation models.

**FIGURE 3:**
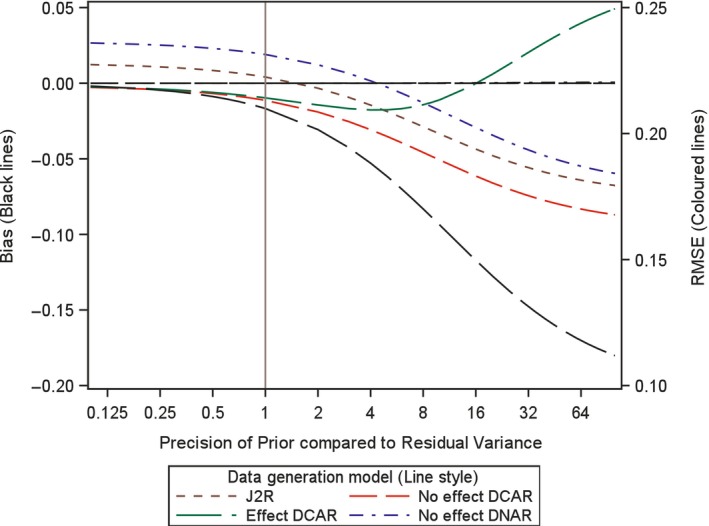
Bias and root mean squared error (RMSE) for retrieved dropout reference‐base centred multiple imputation with *n* = 60 per arm with 40% deviation and 50% missing data in both arms for different dispersion of prior and differing data generation models.

**FIGURE 4:**
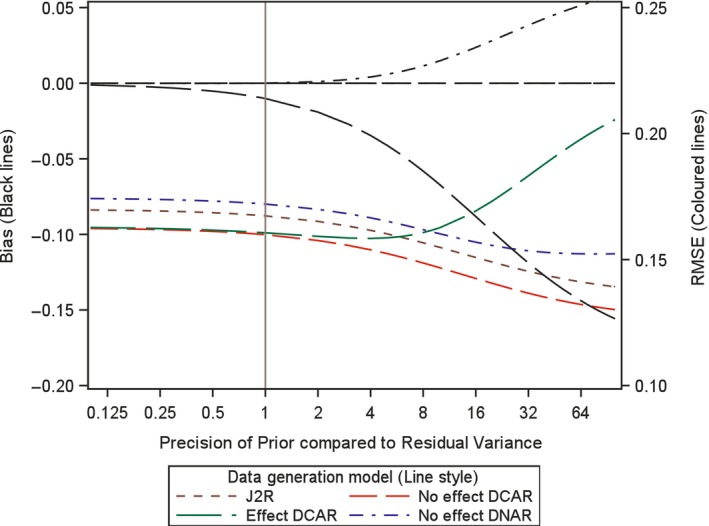
Bias and root mean squared error (RMSE) for retrieved dropout reference‐base centred multiple imputation with *n* = 100 per arm with deviation of 20% in reference and 40% in active, and 50% missing data in both arms for different dispersion of prior and differing data generation models.

**FIGURE 5:**
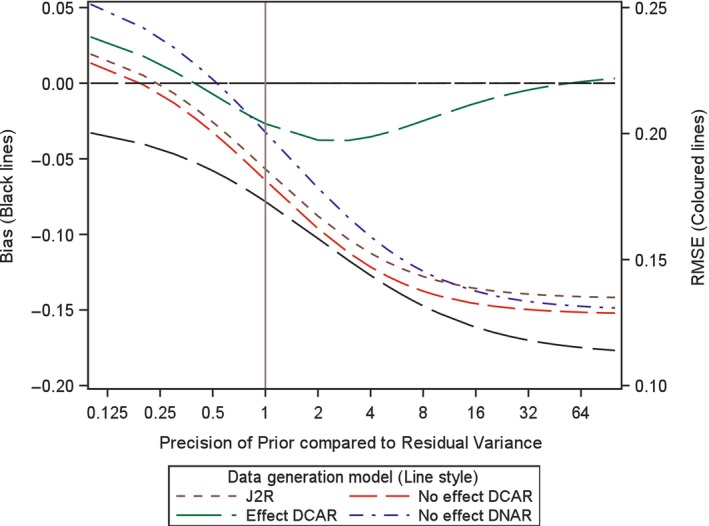
Bias and root mean squared error (RMSE) for retrieved dropout reference‐base centred multiple imputation with *n* = 100 per arm with 20% deviation and 90% missing data in both arms for different dispersion of prior and differing data generation models.

**FIGURE 6:**
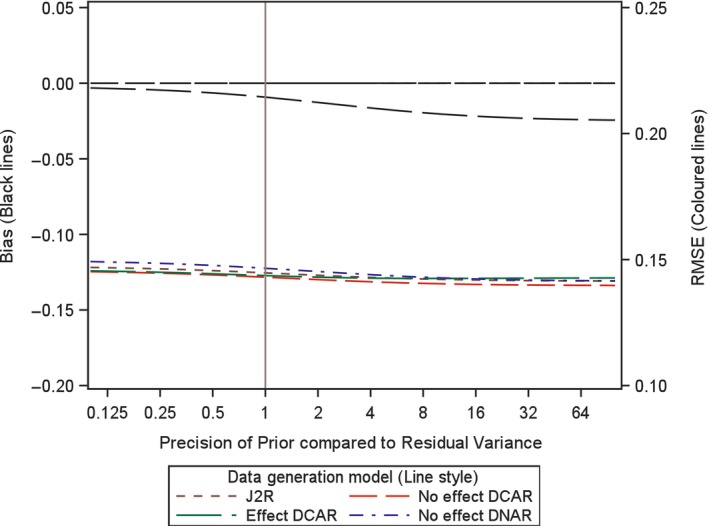
Bias and root mean squared error (RMSE) for retrieved dropout reference‐base centred multiple imputation with *n* = 100 per arm with 5% deviation and 50% missing data in both arms for different dispersion of prior and differing data generation models.

In order to investigate the impact of the choice of variance, or precision, for the prior here we analytically examine the expected bias and root mean square error (RMSE) for the proposed retrieved dropout reference‐base centred multiple imputation method, for different variance values. Investigation is conducted for a two arm trial setting with 100 patients in each arm, no baseline measurement and a single outcome, but now with treatment deviation and missingness in both treatment arms. Appendix 2 shows how the bias and RMSE were generated using analytic results conditional on the numbers in each arm deviating and the number of those with missing data. Then numerical integration was used to average these over the different patterns of deviation and missingness. The combined retrieved dropout reference‐base centred multiple imputation model examined is, $\mu_{\text{miss},0}=\mu_{\mathrm{off},0}=\mu_{\mathrm{on},0}+\gamma_{0}$ and $\mu_{\text{miss},1}=\mu_{\mathrm{off},1}=\mu_{\mathrm{on},0}+\gamma_{1}$ (see Appendix 2), which corresponds to using J2R/CR/CIR as the core model (in this simple setting J2R/CR/CIR equivalent) and for the compliance model using MAR for the missingness process. Four different treatment deviation and missingness settings are considered as follows,

1. 40% deviation in both arms with 50% missingness conditional on deviation (Figures 1, 2, 3).
2. 40% deviation in active arm and 20% deviation in placebo arm and with 50% missingness conditional in both arms (Figure 4).
3. 20% deviation in both arms with 90% missingness conditional on deviation (Figure 5).
4. 5% deviation in both arms with 50% missingness conditional on deviation (Figure 6).

For setting 1 (40% deviation in both arms with 50% missingness), we additionally explored this with 60 patients, then 200 patients per arm. In each setting, we plot the bias and the RMSE for the treatment estimate against the precision of the prior relative to that for the residual variance of the outcome. This is done for four different underlying data generation mechanisms which was achieved by considering data with different values for the four means ($\mu_{\mathrm{off},0},\mu_{\mathrm{on},0},\mu_{\mathrm{off},1},\mu_{\mathrm{on},1}$) and the two associated gamma parameters ($\gamma_{0}$ and $\gamma_{1}$) for each treatment arm (i.e., assuming data with different departures between the core and compliance model, values described below). It is not necessary to simulate data but rather derive exact results conditional on pattern of deviation and missingness and numerically summarise these across the patterns, as explained in Appendix 2. The residual variance is set to 1 in the generated data so the precision of the prior can be seen as the number of new data points that are effectively being added over and above an uninformative prior. Specifically, we explored each of the following four data generation scenarios, based on different configurations of means for the data distribution (as shown in Figures 1, 2, 3, 4, 5, 6 using separate line styles). The graphs show exact results (no simulation error and based on an infinite number of imputations) estimated at over 30 values for the precision of Prior, with a smoothed line drawn through the points, First, no TE at all and deviation completely at random, where all four means are set to a nominal value of 1 ($\mu_{\mathrm{off},0}=1,\mu_{\mathrm{on},0}=1,\mu_{\mathrm{off},1}=1,\mu_{\mathrm{on},1}=1$) and $\gamma_{0}=0,\gamma_{1}=0$, labelled No effect DCAR. Second, again similar outcomes between the arms but here the on‐treatment value is 2 ($\mu_{\mathrm{off},0}=1,\mu_{\mathrm{on},0}=2,\mu_{\mathrm{off},1}=1,\mu_{\mathrm{on},1}=2$), indicating that the deviating subjects are scoring less than those on treatment with $\gamma_{0}=-1,\gamma_{1}=-1$, labelled No effect DNAR. Third, a J2R setting where both off‐treatment and reference on‐treatment means remain at one, while only the on‐treatment active mean increases to 2 ($\mu_{\mathrm{off},0}=1,\mu_{\mathrm{on},0}=1,\mu_{\mathrm{off},1}=1,\mu_{\mathrm{on},1}=2$) where $\gamma_{0}=0,\gamma_{1}=0$, labelled J2R. Fourth a deviation completely at random scenario where on and off treatment means are the same with in each arm with value 1 for reference and 2 for active ($\mu_{\mathrm{off},0}=1,\mu_{\mathrm{on},0}=1,\mu_{\mathrm{off},1}=2,\mu_{\mathrm{on},1}=2$) and $\gamma_{0}=-1,\gamma_{1}=-1$, labelled Effect DCAR.

In the first setting with 100 patients in each arm and a treatment deviation rate of 40% and a missingness rate of 50% conditional upon having deviated, in both arms, then 20 subjects are expected in each arm who have observed off‐treatment data (20 subjects missing off treatment‐data). In this setting a retrieved dropout compliance model will be easy to fit and the prior should have little impact on both bias and RMSE. We see in Figure 1 the treatment estimate is unbiased irrespective of the prior for three of the scenarios (J2R, No effect DCAR, No Effect DNAR). This is the same for smaller and larger sample sizes (see Figures 2 and 3). For Effect DCAR, the non‐zero gamma values in the data generating model conflict with the prior, generating an increase in bias as the prior gains precision. This illustrates how when the core model is wrong strong priors can induce significant bias. The other scenario with non‐zero gamma, No effect DNAR, has zero bias due to the symmetry of the two arms, rather than the appropriateness of the core model as for J2R and No effect DCAR. This is clear in Figure 4 where the deviation rate is changed from 40% to 20% in the reference arm (remaining at 40% in the active arm). With sufficient off‐treatment data to estimate the compliance model and with a relative precision of one for the prior (Figures 1, 2, 3, 4), the proposed retrieved dropout reference‐base centred multiple imputation method has little impact on bias and RMSE, even with a badly chosen core model when the data is Effect DCAR. Overall, with 20% to 40% deviation and 50% missing data, when we can trust the core model (i.e., where gamma is zero), then the precision has little impact. If the core model is not true then the prior variance may want to be more conservative so not to introduce bias.

When the amount of observed off‐treatment data is small then we expect the prior to have more impact. Figure 5 shows the unlikely situation where the deviation rate is 20% but 90% of these are missing. In this scenario the impact of the precision of the prior on the mean square error is larger for all scenarios. The bias under Effect DCAR is larger and interestingly does not get close to zero for small prior precision. Here the impact of perforated data is becoming apparent. It is this setting where choice of prior becomes important and needs to be based on the plausibility of the core model.

Figure 6 has a lower deviation rate of 5% in both arms, with 50% missing data conditional on deviation. Here we see that when the precision of the prior compared to the residual variance is increased above 1, there is an impact on both the bias and RMSE, but the absolute size of the effect is small as there is little missing data. This illustrates how difficulties only occur when the proportion deviating is large and also the proportion of these with missing data is also large.

## 5 Application to Example Data Sets

When there is sufficient data within patient strata defined by treatment and deviation visit (pattern) then it is relatively easy to define a compliance based multiple imputation procedure within strata that yields a robust estimator of treatment comparison at final outcome. Of course, assumptions about the missingness process conditional upon pattern will remain, a topic that is left until the discussion section. The *historic* and *current* choices for the compliance model offer less robust approaches which can be used with less extensive off‐treatment data.

The *perforated* data set from SWGEMD demonstrates the problems that can occur when exclusively using a compliance based modelling approach. Here with only four visits, seven patients on placebo and six on active deviate between first and second visits. While five out the seven are observed on placebo, none of the six on active are observed (Table 1). This means that the parameters $\gamma_{112}$, $\gamma_{113}$ and $\gamma_{114}$ for the *historic* approach and $\alpha_{12}$ for the *current* approach are not estimable. However, these parameters are in general required for imputation of unobserved values. Rather than supply informative priors for these parameters directly, we proposed above that an extended model should be re‐expressed, as an appropriate RBI core model with extra deviation parameters, which are the difference between the required compliance model and the selected core RBI model. Then mildly informative priors for these deviation parameters are applied which centre on zero. Here we focus on *historic* and *current* as suitable choices for the compliance model used in the extended model. The later requires an assumption that having come off treatment the expected outcome is the same however long ago the deviation occurred. For the RBI core model, we use J2R and CIR as realistic options.

For the antidepressant trial example data, we may expect those on placebo who stop treatment at a specific visit and subsequent follow‐up to possibly

- Fair worse than the placebo mean as they stopped because they are severely depressed, or
- Fair better than the placebo mean as they have improved and see no purpose in remaining in the study, or
- Fair about the same, as the off treatment policy in the trial is the same as the placebo on‐treatment policy.

For the first two the effect may be controlled by regressing on baseline HAMD17 and previous outcome. That is by conditioning on previous depression state the future residuals may be simply MAR. A similar picture may hold for the active arm with the additional consideration of the withdrawal of a potentially active therapy. So some intermediate position between J2R and CIR may be a reasonable choice for the core model. As an example of using a fixed delta as core we show results for an MAR + 2 core where the placebo arm is MAR while the active arm is MAR with 2 units added for each extra visit following deviation, representing a rather pessimistic core.

Using the Wilkinson–Rogers model formulae notation [22], there are two main terms in the model, one for the core model and one for the deviations. Assume the following variables defined at the patient‐visit level; factor Trt is treatment as randomised (two levels here), quantitative indicator OffT with 0 for on‐treatment and 1 for off‐treatment, factor Pattern has value which is the current visit number while on treatment and the index of last on‐treatment visit (deviation) when not, factor J2R has as‐randomised treatment level when on treatment and the reference treatment level when off treatment. For more complex cores such as CIR (or LMCF), it is necessary to declare an array of treatment‐by‐visit quantitative variables TrtbyVis [2, 4] reflecting for instance Equation (2) and then regress on these (see Appendix 1 for more detail). Neither of CIR, or (MAR + δ) is nested with the *current* compliance model and both require use of the fuller *historic* extended model, as indicated by blank entries in Table 4.

The core part of the model is merely J2R * Visit for J2R or TrtbyVis1‐TrtbyVis8 for CIR. The deviations are OffT * Trt * Visit * Pattern for *historic* or OffT * Trt * Visit for *current*. Then any baseline covariates such as Baseline can be added as required, such as in this complete model

$$\;J2R^{*}\text{Visit}\;+\;{\text{OffT}}^{*}{\mathrm{Trt}}^{*}{\text{Visit}}^{*}\text{Pattern}\;+\;{\text{Baseline}}^{*}{\text{Visit}}^{*}\mathrm{Trt}$$

Note here how both second and third terms are regressions on a quantitative variable, OffT or Baseline. Importantly when a patient is still on treatment OffT is zero and the model reduces to J2R * Visit + Baseline * Visit * Trt. This requires that OffT is not treated as a factor (not on the CLASS statement in SAS).

Using the SAS procedure proc BGLIMM this Bayesian model can be implemented directly allowing for missing data. In the discussion we consider alternatives to using SAS proc BGLIMM for implementation. The repeated measures multivariate Normal is specified through the REPEATED statement usually using an unstructured covariance matrix which could be grouped by treatment arm if required. As long as conjugate priors are used, as here, the procedure uses direct sampling resulting in very little serial correlation in the sampled parameters. However, to take advantage of the proposal, mildly informative priors need to be attached to the $\gamma^{*}$ or $\;\alpha^{*}$ parameters. The names automatically allocated to the deviation parameters by the SAS procedure are long and include the variable name and associated value for each element of associated term, for instance ‘OffT * Trt 1 * visit 2 * Pattern 1’. The example code attached to this paper demonstrates how this can be accomplished most easily. Numeric factor values make this easier, but care must be taken as SAS names can be no longer than 32 characters. Three template sets of SAS code that generate the results in Table 5 for the *covered* data set using a variance of 40 for the priors and the three approaches, J2R‐*historic*, J2R‐*current* and CIR‐*historic* are available directly [23] or from SWGEMD [24]. They are based on pure SAS code and do not include external macro calls, allowing easy validation using standard one‐off validation protocols for user‐written SAS code.

**TABLE 5:** Mean treatment policy difference and SE using MI for two similar data sets using differing methods.

Method	*Covered*				*Perforated*			
Reference‐based MI	Mean	SE	Mean	SE	Mean	SE	Mean	SE
J2R only	2.18	1.13			2.17	1.13		
J2R + observed Off‐treatment data	2.28	1.05			2.39	1.05		
Compliance‐based MI	*Historic*		*Current*		*Historic*		*Current*	
Outcomes missing for patient‐visits with non‐estimable imputation parameters^a^	2.32	1.10	2.34	1.07	2.45^a^	1.08^a^	2.74^a^	1.05^a^
Non‐estimable imputation parameters set to zero^b^	2.32	1.10	2.34	1.07	2.44^b^	1.10^b^	2.39^b^	1.06^b^
Priors for non‐estimable imputation parameters using Bayesian MVN model^c^	2.31	1.10	2.35	1.06	2.75^c^	2.39^c^	2.52^c^	1.05^c^
J2R with *historic* and *current* extensions with varying prior variance								
Var = 1	2.28	1.05	2.29	1.05	2.38	1.04	2.42	1.04
Var = 10	2.31	1.06	2.33	1.06	2.40	1.08	2.49	1.05
Var = 40	2.32	1.08	2.35	1.06	2.41	1.16	2.51	1.05
Var = 160	2.32	1.09	2.36	1.06	2.44	1.45	2.52	1.05
Var = 1000	2.32	1.10	2.36	1.06	2.63	2.80	2.52	1.05
CIR with *historic* extension with varying prior variance								
Var = 1	2.41	1.04			2.42	1.04		
Var = 10	2.39	1.06			2.41	1.08		
Var = 40	2.36	1.08			2.41	1.16		
Var = 160	2.34	1.09			2.44	1.45		
Var = 1000	2.32	1.10			2.63	2.79		
MAR + 2 with *historic* extension with varying prior variance								
Var = 1	2.53	1.06			2.43	1.07		
Var = 10	2.44	1.07			2.31	1.09		
Var = 40	2.38	1.08			2.24	1.17		
Var = 160	2.34	1.09			3.24	1.48		
Var = 1000	2.33	1.10			2.43	2.81		

^a^ Those patient visits which cannot be imputed are removed. Implemented using %MISTEP macro in SAS.

^b^ In SAS Proc MI sets un‐estimable parameters to zero before imputing.

^c^ Prior *N*(0, 1000) used for all fixed effects as some parameters non‐estimable, implemented using Proc BGLIMM in SAS.

Rather than build the imputed data set in a separate stage, we note that the required values have already been calculated as part of the MCMC process as the estimated missing data parameters. The BGLIMM procedure makes these available as a sample from their posterior distribution. These are merged back with the original data to form the imputed data sets, one based on each sample from the posterior. This is not necessary and a separate prediction stage could be used.

Using the residual variance of the outcome at final visit from a simple MMRM model of on‐treatment data (as described in Section 4.3), for the example data sets used here we chose the variance to be 40. This value could alternatively be chosen during trial design based on previous experience and written into the protocol or analysis plan. Here we show results for these two data sets with the variance for prior changing through 1, 10, 40, 160 and 1000 for each of a range of choices for model alongside results using classic J2R RBI using proc BGLIMM (with or without observed off‐treatment data added) and the standard *historic* and *current* compliance models without re‐parameterisation using three differing computational methods (proc MI, MISTEP macro or proc BGLIMM). For the *perforated* data set the latter three computational routes give different answers based on how they cope with the non‐estimable parameters. For the SWGEMD proc MI route any non‐estimable parameter value is set to zero in the imputation stage. In the MISTEP macro it effectively removes any affected patient‐visit combination by setting the imputed value to missing. For the BGLIMM route using MCMC and Bayesian parameters to represent missing values, we use non‐informative priors for all parameters. This should be the most robust route. We used 10,000 imputations which leads to an approximate Monte Carlo SE of 0.002 for treatment difference, while our results are presented to 2 decimal places. For all multiple imputation methods, results were combined across imputed data sets for inference using Rubin's rules.

The imputation model has visit‐by‐baseline added in every case. The analysis is ANCOVA based on the change in HAMD17 at Visit 6 with randomised treatment and baseline as the only covariates.

### 5.1 Results

The results are summarised in Table 5. The left side uses the *covered* data set where all parameters in the *historic* and *current* models can be estimated, while the right uses the *perforated* data set where difficulties in model fitting occur. The top two rows show the simplest approach using the RBI method J2R using on‐treatment data, either on its own or with observed off‐treatment data added back in.

When J2R MI is implemented for the *covered* data set, adding the observed off‐treatment data back to replace the imputed J2R data when available changes the estimated mean difference from 2.18 to 2.28 and reduces the standard error from 1.13 to 1.05 as expected. This reflects how the ongoing treatment difference is partially maintained after deviation in the observed, which contrasts with J2R where it is assumed the TE immediately disappears following deviation.

For the *perforated* data set the pattern in SE is similar but the mean difference increases by a larger factor from 2.17 with J2R MI only to 2.39 with J2R MI and observed off treatment data. This is because the placebo arm has more off‐treatment observed data and less observed on‐treatment data in the active arm (Table 1), which increases the overall treatment‐policy difference compared to the *covered* data set.

For the compliance based MI analysis, as we expect, when all parameters in the *historic* and *current* compliance models are estimable the differing computational routes deliver the same result. For the compliance based MI analysis using the *covered* data set the simpler *current* model gives a smaller SE of 1.07 (or 1.06) compared to the fuller *historic* at 1.10 with a small change in mean from 2.34 (or 2.35) to 2.32 (or 2.31) when all parameters are estimated. For the *perforated* data set all three computational methods have to take evasive steps for both the simpler *current* and the *historic* model. The stepwise approaches that either do not impute missing outcomes for patient‐visits with non‐estimable imputation parameters or set non‐estimable imputation parameters to zero fair worst as they need to impute at the intermediate Visit 2 where the non‐estimable parameter is required. The former approach replaces any patient‐visit combinations which cannot be imputed by missing values effectively removing the patient from the analysis, and the mean becomes biased while the SE gets smaller showing how off‐treatment patients increase variability in the outcome variable for the merged treatment‐policy data set. That is those on and those off treatment at final visit are inherently different and reducing the number in the smaller subgroup decreases the variance in the merged set. The second procedure effectively sets the non‐estimable $\alpha_{12}$ to zero and carries on including the patient in the analysis imputing as if they had complied. This is safe as it allows the imputation to proceed to subsequent visits with a full set of patients. But it makes an inherent assumption while most likely underestimating the SE as it ignores the lack of compliance. The same picture applies to the third computational approach where all fixed effect parameters are given a broad prior with variance of 1000, with the SE increasing in the *historic* case where the non‐estimable parameter $\gamma_{124}$ is required directly for imputation at the final visit. Diagnosis of worm plots from the non‐estimable parameters indicates roaming during MCMC. For the simpler *current* model the third computational approach results (implemented using proc BGLIMM) are valid and match the later results using the recommended route.

The extended core model using J2R as core with differing variances for priors behaves well with both data sets. In the extreme with unit variance it behaves like J2R RBI with added on‐treatment data, while with larger prior variance for the deviations it behaves like the associated off‐treatment model *historic* or *current*. With the *perforated* data set the SE goes up as the variance goes up for J2R + *historic*, but remains stable for J2R + *current*. With large variance for prior J2R + *historic* becomes unstable just like the *historic* compliance model.

The CIR core model is only nested within the full *historic* compliance model and should not be used with the *current* compliance model. This is because the impact of having deviated under CIR depends upon the visit at which the patient deviated. Only results using the *historic* approach are presented. Using CIR rather than J2R as core model is expected to increase the estimated treatment difference as the TE from the deviation visit is carried forward. With the *covered* data set this is evident when a stronger prior is used such as unit variance. With our recommended mild prior variance of 40, the increase with CIR is 2.36 compared to 2.32 for J2R. For the *perforated* data set it should be remembered that large variances for the priors will lead to problems with the MCMC chain and so only variances 1–160 are of interest. With prior variance of 1000 the SE is large while the non‐estimable parameter roams in the MCMC chain. Using the recommended variance for prior of 40 the results for CIR + *historic* model are very similar to those for J2R + *historic*.

When the core model is MAR with a delta of +2 for each subsequent visit after deviation, the mean treatment difference is 2.38, which is larger than for either J2R or CIR. This reflects a strong deleterious impact of withdrawal of active treatment in the core model.

## 6 Discussion

In this article we have considered the analysis of clinical trials with quantitative longitudinal outcome data, where not all patients remain on treatment and complete follow‐up to the end of the study and a treatment policy strategy is of interest for addressing treatment withdrawal. Retrieved dropout multiple imputation using off‐treatment data (also referred to as compliance‐based multiple imputation) has been recommended for estimation in this setting. However, a perforated data structure may render such imputation methods infeasible since imputation model parameters may be inestimable. With several follow‐up time points a perforated data structure becomes increasingly likely. We have proposed a novel imputation model parameterisation for retrieved dropout multiple imputation, centred on a core reference‐based model, which can be implemented with limited observed off‐treatment data.

In previous work others have proposed the use of retrieved drop out multiple imputation methods as preferable in this setting and where not possible, due to convergence issues, reverting to simpler multiple imputation methods such as the RTB method or the washout method [2] or reference‐based multiple imputation [3]. Our method improves upon the latter fall‐back approaches by utilising partially observed off‐treatment data combined with a reference‐based model. Appealingly a single non‐adaptive analysis plan can be presented when rates of retrieved off‐treatment data are unknown. This may be preferred to an adaptive analysis approach dependent on the realised data structure, which may be sub‐optimal. Any adaptive analysis that switches from a retrieved dropout compliance model to an RBI model will involve an effective step even when using off‐treatment reference arm data in the parameter estimation stage [5]. In contrast the proposed method will smoothly transition between the two approaches as the off‐treatment parameters get less well estimated. Both approaches require a unique aspect of the analysis strategy to be predefined. For an adaptive analysis plan, it is the rules, while for the proposed approach it is the priors' variances for the additional parameters.

It has previously been shown that reference‐based multiple imputation using Rubin's rules provides approximately information anchored inference [25]. That is, Rubin's variance estimator for the TE ensures that the loss of information due to missing data is approximately the same as analysis under the MAR assumption for a broad range of commonly used reference‐based alternatives. It has also been shown that delta‐based multiple imputation with a fixed delta is information anchored; but when an additional prior is incorporated on delta the variance of the MI TE, estimated using Rubin's rules, will incorporate the additional variance on delta (because the variation in delta increases the between‐imputation variance) [25]. Since the proposed method consists of a reference based model plus some additional model parameters with a prior mean of zero and specified variance, it can be inferred that when there is little observed off‐treatment data and the imputation behaves similar to reference‐based multiple imputation, the variance estimator for the TE will be approximately information anchored with an additional increase in variance, that is, additional loss of information that is dependent on the prior variance assumed for the extended model parameter. When there is a large proportion of observed off treatment data the imputation will behave like the chosen‐off‐treatment compliance model, and the variance estimator for the TE will behave more similarly to that seen in retrieved dropout multiple imputation. Thus, there will be some bias‐variance trade off, dependent on the amount of observed off‐treatment data. Our results in Table 5 show how increasing the prior variance for the extended model increases the variance for the estimated TE by a small amount. It has also previously been shown using Rubin's variance estimate in reference‐based multiple imputation overestimates the empirical repeated sampling variance of the reference based TE [25]. This leads to type I error being controlled at levels below 5% under the null. A more extensive simulation study is now required to further explore the properties of Rubin's variance estimate, type I error in this setting and the bias variance trade off with different choices for the prior variance and is now the focus of further work.

Our initial analytical investigations here showed when we can trust the core model (i.e., where gamma is zero), then the precision of the prior's variance has little impact on bias and RMSE. If the core model is not believed to be true then the prior variance may want to be more conservative so not to introduce bias, and when the amount of observed off‐treatment data is small then we expect the prior variance to have more impact. However generally with little missing data the impact of the prior variance will be small. More extensive simulation studies should investigate the methods performance under different conditions including for differing amounts of off‐treatment data when the reference‐based assumption is or is not correct and the impact of different priors and trial sample sizes. Simulation studies are also required to compare the proposed approach to the established ones of reference‐based multiple imputation and retrieved drop out multiple imputation under such different conditions to fully understand the individual settings in which each method has preferred performance.

If the deviation process is not at random, then it is still possible by using off‐treatment data to make an unbiased estimate of the treatment policy estimand. However, if the missingness mechanism is not MAR then other approaches are needed to generate the missed data. The main driver for such MNAR data could be that those who immediately leave the study are in some way different from those remaining. By conditioning on proxies for these factors the MAR approximation should improve. The MAR assumption at trial withdrawal can be made more likely by including covariates that tell us about treatment deviation (e.g., reason for withdrawal) or by stratifying the compliance model by treatment. Or a delta‐based multiple imputation approach may be useful. Here delta is applied following trial withdrawal rather than in the core model which handles post‐deviation irregularity.

We have used an open access part computer generated data set to demonstrate how to implement the proposed method and enable readers to replicate. As such it is important not to draw conclusions from the data about the types of pattern that can be expected in terms of treatment withdrawal and/or study withdrawal. It is important that therapeutic areas make publicly available summary data on the ability to collect such off‐treatment data and the likely trajectories for those who do not comply.

In these example data sets all those patients who come off treatment either immediately stop further observation or are observed right to the end of the study. There are no patients with some but only some of their follow‐on data available. Patients who have some but not all post‐deviation data will have imputation conditioned on both their on‐treatment and their off‐treatment data. This will generate more complex scenarios than those faced in our two examples. For the *current* approach the fact that the $\alpha_{12}$ parameter is not estimable is unimportant in our examples as it is not actually used for imputation. Patients who need imputation at the final visit have no off‐treatment data and so knowing the mean at Visit 2 is not needed. However, if there were patients who deviate and have only some off‐treatment data then knowing the value for $\alpha_{12}$ would be potentially important. The basic idea of providing a mildly informative prior for the deviations from a core RBI model should be even more useful in the more realistic setting of partial off‐treatment data within patient.

For the example data sets we explored J2R, CIR and MAR + 2 as core models. As noted in Section 4 other models can be used for the core model. We also explored the historic (OffT * Trt * Visit * Pattern) and current (OffT * Trt * Visit) compliance models for the example data sets. One is not restricted to these choices. For example, as suggested by a reviewer of this paper one could also consider a compliance model with a term ‘OffT * J2R * Visit * Pattern’ instead of ‘OffT * Trt * Visit * Pattern’ (i.e., pooling data across arms in the off‐treatment period). This would change the resulting compliance model so there is no TE after deviation, so both arms have same off‐treatment means, but this is different from reference on‐treatment mean at that visit. This might be particularly useful for studies of symptomatic treatments with a very short‐lived effect.

We have assumed there are no interim missing data although we can reasonably expect to experience such issues in practice. Both on‐treatment and off‐treatment intermediate missing data could be handled with MAR Multiple Imputation. This is automatic in the associated BGLIMM code when the response is set to a missing value. When data are missing for other reasons beyond the intercurrent events handled by use of treatment policy estimand, such as death, other strategies may be needed. Multiple imputation under other scenarios may allow a joined‐up approach to the disparate types of intercurrent event. Where all subsequent patient data is missing while on treatment, such as when analysis is performed before every patient completes the full study period, it may be necessary to impute treatment withdrawal events using logistic regression alongside imputation of outcome using an alternating stepwise approach. Otherwise, bias may be introduced by a lack of treatment withdrawals being identified.

We have assumed independence of the priors for the deviations. If there are several visits within a study, we may expect the deviations to be in the same direction at adjacent visits. So one might want to build in some positive correlation between adjacent deviations within treatment. This can be done within the code we propose, by adding extra correlation rows to the priors data set. A correlation as large as 0.5 might be suitable, but we have not explored this yet.

Rather than using BGLIMM, the *historic* approach can be implemented in a stepwise way visit by visit using standard Bayesian univariate regression. Such software is generally available and can also be programmed oneself as the required priors are all conjugate leading to direct sampling rather than involving complex MCMC algorithms. One advantage of the marginal BGLIMM approach is that it can automatically handle intermediate missing values under MAR. An advantage of a sequential implementation is that the RBI idea of switching covariance matrix at deviation [3] could be implemented by stratifying at each visit the regression on previous observed values by whether the observed value was—or off‐treatment. For the *current* approach the Bayesian univariate regressions need to be based on previous residuals. Other than BGLIMM, the only SAS Bayesian regression procedure which allows user‐specified priors is GENMOD. However, the BY statement in this procedure currently uses the same seed for each BY group, so messy macro looping is required. Also, the imputed values are not directly available as in BGLIMM.

A similar approach could be used for other types of outcome. Both RBI [26] and compliance [14] models have been proposed for recurrent events based on an extended Negative Binomial, allowing RBI to be nested within the compliance model. Roger et al. [14] suggest two computational routes, the second of these a gamma‐Poisson log‐linear model across multiple periods of patient‐specific fixed length could be implemented in BGLIMM making this an easily programmable option. A binary outcome could be handled in a similar fashion. For time‐to‐event outcomes Atkinson et al. [27] and Jin [28] both propose RBI models in a proportional hazards setting before imputation. These could be used in this way, nesting RBI within a selection model. But easy computational routes are not readily available. However, interpretation of information borrowed between arms is complicated by the differing populations at risk due to ongoing patient selection, a commonly stated limitation of the proportional hazards approach. A nested approach based on a restricted mean survival time (RMST) regression model might be possible, but this area is not well explored.

As alluded to in the introduction, the proposed approach could similarly be used to handle missing data following different types of intercurrent events using a treatment policy strategy, such as temporary treatment interruptions or use of rescue medications. Such events could require more complex modelling scenarios than those explored within this manuscript requiring careful model parametrisation, also dependent on whether those patients who temporarily interrupt treatment (or use rescue medication) either immediately stop all further observation or have any subsequent off‐ and/or on‐treatment follow‐up. This would be interesting to explore in further work.

Overall, we have illustrated how Multiple imputation provides a computationally practical method for inference in this framework. We propose that a core reference‐based model is combined with a retrieved dropout compliance model, using both on‐ and off‐treatment data to form an extended model for imputation. SAS code has been provided to readily enable implementation of the proposed method.

## Ethics Statement

The authors have nothing to report.

## Consent

The authors have nothing to report.

## Conflicts of Interest

James Roger is a previous employee of GSK, J&J. Livedata Process and Livedata (UK) Ltd; he is a shareholder in GSK and his wife is a shareholder in AZ and GSK.

## Data Availability

The data analysed in this article are open access and available at https://www.lshtm.ac.uk/research/centres‐projects‐groups/missing‐data#dia‐missing‐data [9].

## Appendix 1

### 1.1

This appendix shows how to implement this approach in SAS and generate an Imputation data set, ImputedData.

- Generate a vertical data set InputData with the required variables.
  It is assumed that the data set is in vertical format (One patient‐visit per record) already indexed by patient identification, Patient, and visit number Visit. Also that there are two linked variables, variable OffT taking values 0 or 1, where 1 indicates the patient has come off treatment and LastVis which is the visit index of the patient's last on‐treatment visit. The LastVis value relates to the patient and has the same value for all records within a patient, while the OffT is a numeric indicator variable which varies from visit to visit following the definition.
  if Visit > LastVis then OffT = 1; else OffT = 0;
  The variable Pattern is a derived factor also defined at the visit level taking the current visit index while still on treatment and remaining at the index value of the last visit on‐treatment for that and all remaining visits.
  if Visit > Lastvis then Pattern = Lastvis; else Pattern = Visit;
  Also the variable Trt holds the treatment levels, 0 and 1 here, and J2R is a factor holding the treatment level while on treatment and the reference level 0 while off treatment.
  if Trt = 0 then J2R = 0; else if visit<=Lastvis then J2R = 1; else J2R = 0;
- Here we fit the Bayesian model with Change as the outcome variable and baseline covariate Basval using J2R + historic as method. One thousand samples are created with a thinning of 5. Include the plots = trace option to see diagnostic plots for the MCMC process.
  proc bglimm data = InputData seed = 21 outpost =  MyPosterior nmc = 5000 thin = 5;  * plots = trace;
  class Patient Trt Visit J2R Pattern;
  model Change = OffT * Trt * Visit * Pattern J2R * Visit Basval * Visit/noint CPRIOR = NORMAL(Input = MyPriors);
  repeated Visit/subject = Patient type = un;
  run;
  If a ‘delta’ approach is required for the core model then this can implement using the OFFSET option on the model statement, along with a variable holding the required delta values. But beware that the BGLIMM procedure may need a Hot Fix for this to give correct values. A workaround for this when using the identity link is to subtract the offset from the response variable before running BGLIMM and then add it back to the imputed values.
- The priors for the parameters in the term OffT * Trt * Visit * Pattern need to have been set in the data set MyPriors, and running the BGLIMM procedure as above without the CPRIOR = option will provide their names. Then code like this can be used to set the zero mean and variance at 40 thus.
  options validvarname = any;
  Data Mypriors;
  length _Type_ $4;
  %macro dummy();
  %do i = 0%to 1; %do j = 1%to 4; %do k = 1%to &j;
  “OffT * Trt &i. * visit &j. * Pattern &k” n = 0;
  %end; %end; %end;
  _Type_ = “Mean”; output;
  %do i = 0%to 1; %do j = 1%to 4; %do k = 1%to &j; ‘OffT * Trt &i. * visit &j. * Pattern &k’ n = 40;
  %end; %end; %end;
  %mend dummy; %dummy;
  _Type_ = ‘Var’; output.
  run;
  The validvarname option is required so that the variable names can include the spaces required by BGLIMM. The dummy macro is required to generate the names as the SAS language does not allow macro do loops within open code.
- Then the 1000 sampled posterior values for the missing data are merged back with the original data to create 1000 imputed data sets. This can be done using the %BGI macro from the DIA website or if required use code generated by the %BGI macro as template code to directly carry out a transpose of the MyPosterior data set and then merge it back into the original data set. The required call to the macro is
  %BGI(Data = InputData, Post = MyPosterior, Out = ImputedData, Response = Change);
- For other core models it is necessary to set up a treatment by visit array TrtbyVis in the InputData data set to specify the required RBI method. First declare the array and zero the elements.
  array TrtbyVis [2,4];
  do t = 1 to 2; do v = 1 to 4;
  TrtbyVis[t,v] = 0;
  end; end;
  then for CIR use, …
  if Trt = 0 then TrtbyVis[Trt + 1,Visit] = 1;
  else if Visit<=Lastvis then TrtbyVis[Trt + 1, Visit] = 1;
  else do;
  TrtbyVis[1,Visit] = 1;
  TrtbyVis[Trt + 1, Lastvis] = 1;
  TrtbyVis[1,Lastvis] = −1;
  end;
  or for LMCF use …
  if Visit<=Lastvis then TrtbyVis[Trt + 1,Visit] = 1;
  else TrtbyVis[Trt + 1,Lastvis] = 1.
  and one could do J2R this way thus …
  if Trt = 0 then TrtbyVis[Trt + 1,Visit] = 1;
  else if Visit<=Lastvis then TrtbyVis[Trt + 1,Visit] = 1;
  else TrtbyVis[1,Visit] = 1;
  end;
  and finally replace J2R by TrtbyVis1‐8 in the model statement.
  model Change = Offt * Trt * visit * Pattern TrtbyVis1-TrtbyVis8 Basval * Visit/noint.

## Appendix 2

### 2.1

In this appendix, we extend the simple illustration introduced in Section 4.1, by allowing for withdrawal and missingness in the reference arm as well as the active arm. The simplicity of the illustration allows direct calculation of the impact of the priors in the Bayesian modelling stage of the multiple imputation. But the setting is complex enough to help identify the impact of mildly or strongly informative priors. The notation is the same but with the addition of rates $p_{0}$ and $q_{0}$ for withdrawal and missingness in the reference arm and count $n_{j,0}$ and population mean $\mu_{j,0}$ for j=miss,off,on, rather than just $n_{0}$ and $\mu_{0}$. The number in each arm, $N_{0}=\left(n_{\text{miss},0}+n_{\mathrm{off},0}+n_{\mathrm{on},0}\right)$ for reference and $N_{1}=\left(n_{\text{miss},1}+n_{\mathrm{off},1}+n_{\mathrm{on},1}\right)$ for active, are fixed throughout. The contrast of interest is

the difference in expected value for each arm.

#### Fixed Withdrawal and Missingness

There are several layers of random process described and the means and variances $E\left[\;\right]$ and $V\left(\;\right)$ are subscripted to indicate the layer:

- *Y*, for observed data, usually used conditional on the numbers of on, off and missing patients in each arm.
- Post, for Bayesian posterior conditional upon observed data.
- Imp, for summation across imputations which are assumed infinite in number, while also sampling from the posterior.

Outside these there is the withdrawal and missingness processes summarised by $p_{0}$, $q_{0}$, $p_{1}$ and $q_{1}$. In the next section, this later layer is handled by direct enumeration. This section assumes the numbers withdrawing and missing in the two arms is fixed.

This is a very simple model for compliance allowing for missing data while off‐treatment with three states, on‐treatment, off‐treatment and lost‐to‐follow‐up with a single observation and no baseline information. Patients are assumed independent, so that conditional on which of the three groups a subject falls into his outcome is sampled from a normal distribution with mean that depends on the treatment arm and whether the subject is observed and on or off treatment. That is conditional on the number observed/withdrawn there are six means $\mu_{\text{miss},0},\mu_{\mathrm{off},0},\mu_{\mathrm{on},0},\mu_{\text{miss},1},\mu_{\mathrm{off},1},\mu_{\mathrm{on},1},$ and without further constraint $\mu_{\text{miss},0}$ and $\mu_{\text{miss},1}$ are not estimable. An obvious simple compliance model in this setting sets $\mu_{\text{miss},0}=\mu_{\mathrm{off},0}$ and $\mu_{\text{miss},1}=\mu_{\mathrm{off},1}$ where off‐treatment mean is the same whether observed or not, assuming MAR for the missingness process. Then we can estimate all six means as long as the counts $n_{\mathrm{off},0}$ and $n_{\mathrm{off},1}$ are greater than zero whenever the $n_{\text{miss},0}$ or $n_{\text{miss},1}$ for the same arm is greater than zero. Under this MAR assumption

A RBI J2R model uses $\mu_{\text{miss},1}=\mu_{\mathrm{off},1}=\mu_{\text{miss},0}=\mu_{\mathrm{off},0}=\mu_{\mathrm{on},0}$ instead, using the reference arm on‐treatment as mean for the off‐treatment means in both arms. The purpose of this appendix is to investigate the idea of re‐expressing the previous compliance model in the following way, $\mu_{\text{miss},0}=\mu_{\mathrm{off},0}=\mu_{\mathrm{on},0}+\gamma_{0}$ and $\mu_{\text{miss},1}=\mu_{\mathrm{off},1}=\mu_{\mathrm{on},0}+\gamma_{1}$. This is equivalent to the compliance model using MAR for the missingness process. When both gamma parameters $\gamma_{0}$ and $\gamma_{1}$ are fixed at zero then this is the J2R RBI model. If $n_{\mathrm{off},0}=0$ while $n_{\text{miss},0}>0$ then the parameter $\gamma_{0}$ cannot be estimated, while if $n_{\mathrm{off},1}=0$ while $n_{\text{miss},1}>0$ then the parameter $\gamma_{1}$ cannot be estimated. By applying a mild informative prior for $\gamma_{0}$ and $\gamma_{1}$ the six means can all be estimated as long as number observed on treatment in both arms $n_{\mathrm{on},0}$ and $n_{\mathrm{on},1}$ are greater than zero, a very reasonable assumption, leading to an estimate for the required contrast.

The missing data problem is often handled using a multiple imputation approach as detailed in Section 4.2. Rather than jump into an extensive simulation activity at the patient level we explore an analytic approach. First we consider the Bayesian model fit, conditional on a single set of observed data, six counts $n=\left(n_{\text{miss},0},n_{\mathrm{off},0},n_{\mathrm{on},0},n_{\text{miss},1},n_{\mathrm{off},1},n_{\mathrm{on},1}\right)$ assumed fixed at this stage, four observed means ${\bar{y}}_{\mathrm{off},0},{\bar{y}}_{\mathrm{on},0},{\bar{y}}_{\mathrm{off},1}$ and ${\bar{y}}_{\mathrm{on},1}$, and four observed sum of squares about the mean $s_{\mathrm{off},0},s_{\mathrm{on},0},s_{\mathrm{off},1}$ and $s_{\mathrm{on},1}$ all random. We assume independent normal priors for $\mu_{\mathrm{on},0}$, $\mu_{\mathrm{on},1}$, $\gamma_{0}$ and $\gamma_{1}$ with zero means and variances $\sigma^{2}/\psi_{0}$, $\sigma^{2}/\psi_{1}$, $\sigma^{2}/\phi_{0}$ and $\sigma^{2}/\phi_{1}$ (precision $\psi_{0}/\sigma^{2}$, $\psi_{1}/\sigma^{2}$, $\phi_{0}/\sigma^{2}$ and $\phi_{1}/\sigma^{2}$), and uninformative inverse chi‐square prior for $\sigma^{2}$. We will set $\psi_{0}$ and $\psi_{1}$ to zero as uninformative while exploring different values of $\phi_{0}$ and $\phi_{1}$. Such priors give no information about the treatment means on treatment but some about the likely off treatment means compared to those on treatment. Note that $\sigma^{2}$ is not known in advance and this specification is saying that the prior is being defined relative to the unknown residual variance which will be estimated from the data. The technical advantage here is it allows the term to cancel in the definition of the posterior distribution. In practice it has the attribute that if the trial has unexpectedly less precision, larger residual, then the prior is down weighted while if the trial is more precise than expected the precision of the prior is automatically increased.

We stack the four observed means into a single observed vector $Y={\left[{\bar{y}}_{\mathrm{off},0},{\bar{y}}_{\mathrm{on},0},{\bar{y}}_{\mathrm{off},1},{\bar{y}}_{\mathrm{on},1}\right]}^{T}$ and stack the four parameters as parameter vector $\theta ={\left[\mu_{\mathrm{on},0},\gamma_{0},\mu_{\mathrm{on},1},\gamma_{1}\right]}^{T}$. Transpose of vectors and matrices are indicated by suffix capital T. The four means and four sums of squares summarise the data completely conditional upon the number in each group. The mean is

$$E_{Y}\left[Y|n\right]=X\theta$$

where

$$X=\left[\begin{array}{cccc} 1 & 1 & 0 & 0\\ 1 & 0 & 0 & 0\\ 1 & 0 & 0 & 1\\ 0 & 0 & 1 & 0 \end{array}\right]\;\text{and}\;\;X^{T}=\left[\begin{array}{cccc} 1 & 1 & 1 & 0\\ 1 & 0 & 0 & 0\\ 0 & 0 & 0 & 1\\ 0 & 0 & 1 & 0 \end{array}\right]$$

Other core/compliance model combinations will define different matrices X. The observations $Y_{i}$ are independent, so Y is multivariate normal with variance–covariance $\sigma^{2}\Lambda$ where inverse of Λ is

$$\Lambda^{-1}=\left[\begin{array}{cccc} n_{\mathrm{off,}0} & 0 & 0 & 0\\ 0 & n_{\mathrm{on,}0} & 0 & 0\\ 0 & 0 & n_{\mathrm{off,}1} & 0\\ 0 & 0 & 0 & n_{\mathrm{on,}1} \end{array}\right]$$

The prior for θ is normal with zero mean and variance–covariance $\Psi \sigma^{2}$ where inverse of Ψ is

$$\Psi^{-1}=\left[\begin{array}{cccc} \psi_{0} & 0 & 0 & 0\\ 0 & \phi_{0} & 0 & 0\\ 0 & 0 & \psi_{1} & 0\\ 0 & 0 & 0 & \phi_{1} \end{array}\right]$$

The posterior for θ conditional upon $\sigma^{2}$ is normal with mean

$$E_{\text{Post}}\left[\theta |\sigma^{2}\right]={\left(X^{T}\Lambda^{-1}X+\Psi^{-1}\right)}^{-1}X^{T}\Lambda^{-1}Y=\mathrm{ABY}$$

which does not involve the value of $\sigma^{2}$ and variance

$$V_{\text{Post}}\left(\theta |\sigma^{2}\right)=\sigma^{2}{\left(X^{T}\Lambda^{-1}X+\Psi^{-1}\right)}^{-1}=\sigma^{2}A$$

where $A={\left(X^{T}\Lambda^{-1}X+\Psi^{-1}\right)}^{-1}$ and $B=X^{T}\Lambda^{-1}$. The marginal posterior distribution for $\sigma^{2}$ is inverse chi‐square with $E\left[\sigma^{2}\right]=\left(s_{\mathrm{off},0}+s_{\mathrm{on},0}+s_{\mathrm{off},1}+s_{\mathrm{on},1}\right)/\nu$ and ν degrees of freedom, where $\nu =\left(n_{\mathrm{off},0}+n_{\mathrm{on},0}+n_{\mathrm{off},1}+n_{\mathrm{on},1}-4\right)$.

At this stage set $\psi_{0}=\psi_{1}=0$ making the well estimated parameters $\mu_{\mathrm{on},0}$ and $\mu_{\mathrm{on},1}$ have flat priors. This is not required, but makes discussion simpler. We need to invert the matrix A which is possible as long as both $\phi_{0}$ and $\phi_{1}$ and also $n_{\mathrm{on},1}$ but not necessarily $n_{\mathrm{on},0}$ are greater than zero. The value changes from simulated data set to data set, but does not change from imputation to imputation, as the counts n are fixed across imputations.

At each imputation a draw is made from the joint posterior and components of ${\bar{y}}_{\text{miss},0}$ and ${\bar{y}}_{\text{miss},1}$ imputed. These imputed missing values will be normal with means

and

Note how each is a weighted mean across the four observed values Y and is just the observed means off treatment when both $\phi_{0}$ and $\phi_{1}$ are zero.

Their variance and covariance involves the covariance matrix of the posterior of θ and the variance associated with individual imputations involving $\sigma^{2}$.

$$\begin{array}{c} V_{\mathrm{Imp}}\left[\begin{array}{c} {\bar{y}}_{\text{miss},0}\\ {\bar{y}}_{\text{miss},1} \end{array}\right]=\left[\begin{array}{cccc} 1 & 1 & 0 & 0\\ 1 & 0 & 0 & 1 \end{array}\right]A{\left[\begin{array}{cccc} 1 & 1 & 0 & 0\\ 1 & 0 & 0 & 1 \end{array}\right]}^{T}\sigma^{2}\\ \;+\left[\begin{array}{cc} \sigma^{2}/n_{\text{miss},0} & 0\\ 0 & \sigma^{2}/n_{\text{miss},1} \end{array}\right] \end{array}$$

and is undefined when $n_{\mathrm{on},1}$ is zero.

Then we can derive the mean across imputations for the estimated treatment difference *T* based on imputed values, which is our MI based estimate for the contrast of interest, denoted by $\tilde{T}$.

(6)

which is a weighted sum of the four observed means Y with weights $W\left(n\right)$.

It is possible to derive similar exact averages for other statistics conditional upon the pattern of deviation and missingness (the values of n). Two important examples are the variance of the treatment difference in imputed data across imputations and the average across imputations of the estimated variance of the treatment difference estimate. These are components of Rubin's estimate of variance. They and subsequent derived statistics can be averaged across the deviation/missingness pattern in a similar way to that used for the estimate itself in the next section. In this way the choice of prior can be explored on other aspects of the inference process in future. In contrast properties such as the bootstrap estimator cannot be handled in this way as the resampling modifies the deviation/missingness pattern in the resampled data.

#### The Simulation Level

All the material so far is conditional upon the observed vector Y. Now we consider the long term properties across the distribution of Y using numerical integration. Once you have decided on $N_{0},N_{1},\phi_{0}$ and $\phi_{1}$ then as long as you know the random $n=\left(n_{\text{miss},0},n_{\mathrm{off},0},n_{\mathrm{on},0},n_{\text{miss},1},n_{\mathrm{off},1},n_{\mathrm{on},1}\right)$ then the weights $W\left(n\right)=\left(w_{1},w_{2},w_{3},w_{4}\right)$ in Equation (6) are known and fixed. So we can explore the properties of the estimator by using numerical integration over the possible values for n using values based on the withdrawal and missing parameters $p_{0},p_{1},q_{0}$ and $q_{1}$, using postulated means $\tilde{\mu }$. This means that taking expectations over the distribution of the observed data we simply replace the elements in Y by the vector of simulation means for the data $\tilde{\mu }={\left({\tilde{\mu }}_{\mathrm{off},0},{\tilde{\mu }}_{\mathrm{on},0},{\tilde{\mu }}_{\mathrm{off},1},{\tilde{\mu }}_{\mathrm{on},1}\right)}^{T}$ to obtain the simulation mean for $\tilde{T}$ based on Equation (6).

$$E_{Y}\left[\tilde{T}\right]=\sum_{n}P\left[n\right]E_{Y}\left[\tilde{T}|n\right]=\sum_{n}P\left[n\right]E_{Y}\left[W{\left(n\right)}^{T}Y|n\right]=\sum_{n}P\left[n\right]\left[W{\left(n\right)}^{T}\tilde{\mu }\right]$$

where

$$\begin{array}{c} P\left[n\right]=\frac{N_{0}!{\left(1-p_{0}\right)}^{n_{\mathrm{on},0}}{\left(p_{0}\left(1-q_{0}\right)\right)}^{n_{\mathrm{off},0}}{\left(p_{0}q_{0}\right)}^{n_{\text{miss},0}}}{n_{\text{miss},0}!\;n_{\mathrm{off,}0}!\;n_{\mathrm{on},0}!}\\ \;\frac{N_{1}!{\left(1-p_{1}\right)}^{n_{\mathrm{on},1}}{\left(p_{1}\left(1-q_{1}\right)\right)}^{n_{\mathrm{off},1}}{\left(p_{1}q_{1}\right)}^{n_{\text{miss},1}}}{n_{\text{miss},1}!\;n_{\mathrm{off},1}!\;n_{\mathrm{on},1}!} \end{array}$$

The case $n_{\mathrm{on},0}=n_{\mathrm{off},0}=0$ or $n_{\mathrm{on},1}=n_{\mathrm{off},1}=0$ where all data in an arm is missing needs special attention even though the probability may be very small as we use complete enumeration rather than Monte‐Carlo simulation. We ignore the possibilities and reallocate the probability. If the ignored probability is not trivial then bias will be apparent when unexpected.

The actual simulation mean difference is then

allowing the calculation of bias.

More interesting will be the mean squared error of $\tilde{T}$.
